# Potential of Agroindustrial By-Products to Modulate Ruminal Fermentation and Reduce Methane Production: In Vitro Studies

**DOI:** 10.3390/ani12243540

**Published:** 2022-12-15

**Authors:** Carlos Navarro Marcos, Trinidad de Evan, Christian Jiménez, María Dolores Carro

**Affiliations:** Departamento de Producción Agraria, ETSIAAB, Universidad Politécnica de Madrid, Ciudad Universitaria, 28040 Madrid, Spain

**Keywords:** exhausted olive cake, tomato pomace, wine lees, in vitro rumen fermentation, methane

## Abstract

**Simple Summary:**

Exhausted olive cake, tomato pomace and wine lees are agroindustrial by-products extensively produced in the Mediterranean area that could be used in ruminant feeding, but the information on their optimal inclusion level in ruminant diets is scarce. Exhausted olive cake and tomato pomace are fibrous by-products and therefore are more adequate for ruminant feeding, whereas wine lees can have high protein content. The objective of this study was to evaluate the effect of including increasing amounts of these by-products in diets for fattening ruminants on in vitro ruminal fermentation and methane production. We analysed the in vitro fermentation of diets including 0, 6, 12 and 18% of these by-products. Including up to 18% of a 1:1 mixture of exhausted olive cake and tomato pomace had no negative effect on in vitro ruminal fermentation, but decreased ammonia concentrations, probably due to the low crude protein digestibility of both by-products. In contrast, high amounts of wine lees (12 and 18%) had negative effects on in vitro ruminal fermentation, and consequently they could only be included up to 6% to avoid negative effects. The absence of effects of any tested by-product on methane production indicates that they lack antimethanogenic compounds.

**Abstract:**

The effects of including wine lees (WL), exhausted olive cake (EOC) and a 1:1 mixture of EOC and tomato pomace (EOCTP) in diets for fattening ruminants on in vitro fermentation parameters and CH_4_ production were analysed. Ten diets were studied, containing either none of the tested by-products (control), or 6.0, 12.0 or 18.0% of WL, EOC and ECOTP formulated to have similar protein and fiber content. Diets were incubated in vitro with sheep ruminal fluid to measure gas production kinetics and fermentation parameters. Increasing the level of WL, EOC and EOCTP decreased linearly (*p* ≤ 0.009) the potential gas production, but other gas production parameters were unaffected (*p* > 0.05), excepting that EOCTP increased the gas production rate. No differences (*p* ≥ 0.0.05) among diets were observed in total volatile fatty acid (VFA) production at 24 h of incubation for EOC and EOCTP, but NH_3_-N concentration decreased (*p* ≤ 0.003). In contrast, WL at 12.0 and 18.0% decreased (*p* < 0.05) total VFA production and increased the acetate/propionate ratio (*p* < 0.05). None of the by-products had an effect on CH_4_ production (*p* ≥ 0.0.05). Results indicate that EOC and EOCTP could be included up to 18.0% in fattening diets, but lower levels of WL are recommended.

## 1. Introduction

In the last years, the idea of the circular economy has gained importance to satisfy the requirements of sustainable agriculture production, even among policymakers [1]. In fact, in March 2020, the European Commission adopted a new Circular Economy Action Plan within the EU new agenda for sustainable growth. The European transition to a circular economy is aimed to reduce the pressure on natural resources [2], being also essential to achieve the EU 2050 climate neutrality target and stop the loss of biodiversity. As part of this plan, the ‘waste and recycling’ policy aims to protect the environment and human health and to help the European Union transition to a circular economy, while its main targets are to improve waste management, stimulate innovation in recycling, and limit landfilling resources [3]. Utilization of agroindustrial by-products in animal feeding might help accomplish all these objectives, as many of these by-products are very pollutant due to the high water content, and thus are easily putrescible [4,5], making their disposal sometimes challenging. However, many by-products are rich in fiber and are therefore more suitable for ruminant feeding compared to other non-ruminant species, as ruminants are the most efficient digesters of lignocellulose due to ruminal microbiota.

In addition, by-products can contain multiple nutrients and bioactive compounds that could improve the quality of animal products and the health of the animal itself [6], and some of them may be effective at decreasing enteric methane emissions [7,8]. Therefore, increasing the practical use of agroindustrial by-products in ruminant feeding will reduce their negative environmental impact, and might decrease methane emissions associated with the livestock sector which is another proposed objective to fight climate change [9]. The EU produces most of the olive oil and wine of the world (62% and 56%, respectively) [10] and produces up to 29.5% of the worldwide tomato for processing [11]. As a consequence, huge quantities of by-products associated with these industries are generated, such as exhausted olive cake (EOC), tomato pomace (TP) and wine lees (WL).

Exhausted olive cake is a fibrous by-product, rich in polyphenols, which is obtained after chemically extracting the residual oil from olive cake, the main by-product of olive oil production [12]. Tomato pomace is another fibrous by-product that is recovered after processing the whole fruit to obtain tomato paste, and it has a moderate content of crude protein [5]. Wine lees are the waste product that is left at the bottom of the barrel after wine fermentation, and usually are rich in polyphenols [13]. The utilization of these by-products in ruminant feeding can contribute to the sustainability of both agricultural and livestock sectors, but the information on their optimal inclusion levels in diets for fattening ruminants is scarce. Therefore, the objective of this study was to evaluate the effect of including increasing quantities of these agroindustrial by-products, produced in abundant quantities in the EU, in diets for fattening ruminants on the in vitro fermentation parameters and methane production. 

## 2. Materials and Methods

The sheep used as ruminal fluid donors were handled according to the European guidelines for protection of experimental animals. Procedures for ruminal contents sampling were approved by the General Direction of Livestock and Agriculture of the Community of Madrid (Approval number PROEX 035/17). 

### 2.1. By-Products and Diets Formulation

All by-products were obtained from factories located in Spain and their chemical composition is given in Table 1. One sample of red WL was obtained from an organic production winery (Explotaciones Hermanos Delgado; Ciudad Real), in which WL are commercially used to elaborate certified organic concentrates for livestock feeding. The sample of EOC was obtained at an oil extraction plant located in Puente Genil (SACYR Industrial SLU, Spain). This sample underwent an extraction of the residual oil to obtain pomace olive oil, and therefore its ether extract (EE) content was low (Table 1). 

The TP sample had been used in a previous study [5] and it was obtained by pooling 12 TP samples from 2 tomato processing plants (PRONAT and Tomates del Guadiana Sociedad Cooperativa) located in Badajoz (Spain). The effects of including increasing amounts of TP in the diet of fattening ruminants were previously reported by Marcos et al. [5], and therefore were not investigated in this study. A 1:1 mixture of EOC and TP (EOCTP) was prepared to obtain a fibrous ingredient with an average crude protein (CP) content and lower EE content than TP, as high concentrations of EE in by-products could limit their inclusion level in ruminant diets [14].

In addition, samples of common ingredients of ruminant diets (barley, wheat, corn, soybean meal, wheat bran and barley straw) were obtained from a local animal feed factory. All by-products and feeds used to formulate the experimental diets were ground to pass a 1 mm screen using a Retsch ZM 200 ultra centrifugal mill (Retsch GmbH, Haan, Germany) before chemical analyses and in vitro incubations.

One control diet including common feeds in ruminant feeding was formulated to meet the nutrient requirements of fattening ruminants [15]. Nine additional diets were designed to include increasing quantities (6, 12 and 18%, fresh matter basis) of the 3 by-products tested (WL, EOC and EOCTP). All diets containing the by-products were formulated to have CP and neutral detergent fiber (NDF) concentrations similar to the control diet, and their ingredient and chemical composition is shown in Table 2.

The WL partially replaced corn, barley straw and soybean meal, whereas corn, barley straw and wheat bran were partially substituted by EOC in the EOC diets. Finally, the EOCTP mixture replaced increasing amounts of barely straw, soybean meal and wheat bran in the EOCTP diets. The amounts of barley, wheat, calcium soap, calcium carbonate and mineral/vitamin premix remained unchanged in all diets, with the exception of calcium soap that was reduced in the EOCTP diets to keep the EE content below 47.0 g/kg dry matter (DM).

### 2.2. Donor Animals and In Vitro Fermentation of Experimental Diets

The ruminal fluid used as inoculum for the in vitro incubations was obtained from four adult Lacaune sheep (63.5 ± 2.05 kg live weight) provided with a permanent rumen cannula. Sheep were fed a 66:33 grass hay:concentrate diet twice daily, and had free access to fresh and clean water over the trial. The rumen content of each sheep was sampled before the morning feeding, filtered through 4 layers of cheesecloth, and immediately transported to the laboratory into thermal flasks.

The methodology for the in vitro incubation of the experimental diets has been detailed by Marcos et al. [5]. Briefly, 400 mg of each diet were weighted into 120-mL glass vials, which were filled with 40 mL of a 1:4 mixture of ruminal fluid and the culture medium of Goering and Van Soest [16] that was modified to exclude all N-containing chemicals. Modifications consisted in excluding the use of trypticase and replacing the NH_4_HCO_3_ with NaHCO_3_. Vials were capped with rubber stops and incubated at 39 °C for 120 h to measure the gas production kinetics. Identical incubations were performed for 24 h in a different week to measure the main fermentation parameters. Within each incubation run, the ruminal inoculum of each sheep was independently mixed with the culture medium to obtain 4 replicates for each diet. 

In the 120 h incubations two blank vials per ruminal inoculum were included to correct the gas values for the gas produced by the fermentation of endogenous substrates. Gas production was measured at 3, 6, 9, 12, 15, 22, 26, 31, 36, 48, 58, 72, 96, and 120 h using a Delta Ohm DTP704-2BGI pressure transducer (Herter Instruments SL, Barcelona, Spain) and a plastic syringe, and the gas was released after each measurement. In the 24 h incubations, gas production was measured after 8 h of fermentation, and a 15 mL sample was stored in vacuum tubes (Terumo Europe N.V., Leuven, Belgium) for CH_4_ analysis. Immediately after gas sampling the vial content was homogenized, 1 mL was sampled using a syringe and mixed with 1 mL of 0.5 M HCl and immediately frozen (−20 °C) until analysis of volatile fatty acids (VFA) and NH_3_-N concentrations. Finally, after 24 h of incubation samples of both gas and vials content were taken again and processed as described before and the pH was measured using a Crison Basic 20 pH meter (Crison Instruments, Barcelona, Spain). 

### 2.3. Analyses of By-Products and Feeds Chemical Composition and Fermentation Parameters

All analyses were performed in duplicate. Chemical composition of by-products and feeds were analysed following the methodology described by Marcos et al. [5]. In brief, DM, ash and EE were analysed using the Association of Official Analytical Chemists [17] procedures, content of NDF and acid detergent fiber (ADF) were determined according to Van Soest et al. [18], and CP was measured by the Dumas combustion method. Total soluble polyphenols content was analysed in the 3 by-products by the Folin–Ciocalteu assay [19] using an Epoch spectrophotometer (BioTek Instruments Inc., Winooski, VT, USA).

Concentrations of NH_3_-N were determined by the phenol-hypochlorite method [20]. Both VFA and CH_4_ concentrations were analysed by gas chromatography as described by García-Martínez et al. [21] and Martínez et al. [22], respectively.

### 2.4. Calculations and Statistical Analyses

The model Gas = PGP × (1 − e ^(−*c* × (t − *lag*))^) was used to fit gas production data with time by an iterative least squares procedure using the NLIN procedure of SAS [23]. In this model, PGP is the asymptotic gas production, *c* is the fractional rate of gas production, *lag* is the moment when gas production starts, and t is the time of gas measurement. The experimental data fitted well to the model, as the convergence criterion was met on average after 6.25 iterations, all models were highly significant (*p* < 0.001), and the errors for the estimated parameters were lower than 2%. In addition, the average gas production rate (AGPR) was calculated as proposed by France et al. [24]: AGPR = PGP × *c* ÷ [2 × (ln2 + *c* × *lag*)], and the amount of apparently fermented OM (AFOM) was estimated from individual VFA production as described by Demeyer [25].

Data from the diets containing each by-product were analysed independently. Both gas production and fermentation parameters were analysed as a mixed model using the MIXED procedure of SAS [23], in which the effect of the diet was considered fixed and that of the inoculum (ruminal fluid) was considered random. Additionally, polynomial contrasts were used to test for linear and quadratic effects of including increasing quantities of each by-product. When effects were significant, means were compared post hoc using the Tukey’s test. 

In addition, multidimensional scaling analysis was used to compare the fermentation parameters of all diets after 8 and 24 h of in vitro incubation. Euclidian distance was used for ruminal fermentation parameters (VFA and CH_4_ production, NH_3_-N concentrations and AFOM), whereas the Aitchison distance was utilized for VFA molar proportions due to the compositional nature of these data. Differences between diets and inclusion levels were assessed by a MANOVA. These analyses were performed using the core R packages. In all statistical analysis, significance was declared at *p* ≤ 0.05, and *p* values between 0.05 and 0.10 were considered as a trend.

## 3. Results and Discussion 

### 3.1. Fermentation of Diets including Wine Lees

Chemical composition of WL was in the range reported previously by others [26,27], except for the CP content, which was lower. On the contrary, Sato et al. [13] reported lower concentrations of organic matter (OM), CP, NDF, ADF and EE in red WL compared with the present study. The content in TSP of the WL was within the range (17.3–40.9 g/kg DM) reported by Zhijing et al. [28] for red WL, although others have reported much greater values [26]. Chemical composition of WL can be influenced by many factors such as environmental conditions, grape variety and characteristics of the wine making process [29,30]. Moreover, the organic cultivation of the WL used in our study could have contributed to their low CP content, as organic crops usually have lower CP compared with conventional cultivation due to restrictions in N fertilization [31]. 

As shown in Table 3, PGP decreased linearly (*p* < 0.001) as greater WL concentrations were included in the diet, but no other effects on gas production parameters were observed (*p* ≥ 0.0.05). Sato et al. [13] observed that gas production after 12 h of in vitro incubation remained unchanged when rolled barley was replaced with high proportions of WL (up to 22.5% DM), but it was decreased after 48 h of incubation. The lower gas production observed in the late stages of incubation in both studies could be due the lower amounts of fermentable compounds in the WL than in the replaced feeds (mainly corn and soybean meal in our study), as well as to the presence of polyphenols and/or other compounds in the WL that could impair ruminal fermentation [13]. However, the lack of differences among diets in *c*, *lag* and AGPR observed in our study indicates that WL had no detrimental effect on ruminal fermentation during the first fermentation stages. On the contrary, Yao et al. [32] reported that replacing soybean meal with increasing amounts of either fermented or unfermented yellow WL resulted in a linear increase in PGP after 48 h of in vitro incubation with sheep ruminal fluid, even though the in vitro DM and OM degradability was decreased. It should be noticed that protein fermentation generates less gas than carbohydrates fermentation [33], and therefore in vitro fermentation of high-protein feeds, such as soybean meal, can result in lower amounts of gas. Moreover, discrepancies in the results from different studies could also be attributed to the variability of WL composition and to the variable feed ingredients replaced in the diets.

In agreement with the gas production results, including increasing quantities of WL in the diet did not affect (*p* ≥ 0.0.05) either total VFA and CH_4_ production or NH_3_-N concentration after 8 h of fermentation. However, the individual VFA profile was shifted towards more acetate and less propionate (*p* ≤ 0.003), and consequently the acetate/propionate ratio was linearly increased (*p* < 0.001). On the contrary, after 24 h of incubation, WL inclusion linearly decreased (*p* ≤ 0.017) both total VFA production and the amount of AFOM, whereas shifts in the VFA profile remained similar to those observed at 8 h of fermentation. The high content of polyphenols present in WL might negatively affect ruminal fermentation [34], which could partly explain the slightly reduction in VFA production observed after 24 h of fermentation. 

In agreement with our results, Yao et al. [32] reported that replacing increasing amounts of soyabean meal with yellow WL in a 60:40 forage:concentrate diet decreased total VFA production and molar proportions of propionate after 48 h of in vitro incubation, but increased the acetate proportion. Sato et al. [13] also observed similar shifts in the VFA profile after 48 h of in vitro incubation of rolled barley when it was replaced with increasing amounts of WL (up to 22.5%), although the total VFA remained unchanged. The reduced propionate proportions observed in our study at both incubation times for WL12 and WL18 diets were probably due to a reduction in the amount of corn, as starch fermentation in the rumen leads to more propionate production. Yao et al. [32] attributed the lower VFA production observed for the WL-diets to their greater NDF content, but all experimental diets in our study had similar NDF levels (Table 2). As previously discussed, the lower content in high-fermentable compounds in the WL compared with corn and soybean meal can explain the reduced fermentation observed for the WL-diets after 24 h of fermentation. 

Although both Sato et al. [13] and Yao et al. [32] observed that the inclusion of WL in the diet reduced the in vitro NH_3_-N concentrations, no differences (*p* ≥ 0.05) among diets were observed in our study at any incubation time. Sato et al. [13] stated that WL might decrease in the in vitro CP digestibility and NH_3_-N production because of their high tannin concentration. However, the lack of differences in NH_3_-N concentration and the trend (*p* = 0.061) to a linear increase in the proportion of branched-chain VFA (calculated as the sum of isobutyrate, isovalerate and valerate) at 24 h of fermentation when WL were included in the diet indicate that there was no negative effect of WL on dietary CP degradation in our study.

Wine lees are rich in polyphenols and tannins, which might modulate ruminal fermentation, and therefore might affect CH_4_ production [35,36]. Nevertheless, in our study, including WL in the diet had no effect (*p* ≥ 0.0.05) either on CH_4_ production or in the CH_4_/VFA ratio at any incubation time. To our best knowledge this is the first study reporting the lack of antimethanogenic compounds in WL, but other studies have noticed a lack of effects of both red and white grapes on in vitro CH_4_ production [37]. 

### 3.2. Fermentation of Diets including Exhausted Olive Cake

The chemical composition of the EOC was in the range reported by Marcos et al. [12] for EOC samples collected from different olive industries in Spain, although EOC composition is highly variable, being affected by different factors such as the olive cultivar, the characteristics of the fruit, processing techniques and storage conditions [38]. 

Including increasing amounts of EOC in the diet decreased the PGP linearly (Table 4; *p* = 0.003), but the rest of gas production parameters were unaffected (*p* ≥ 0.05). Exhausted olive cake is a highly fibrous by-product with low DM and NDF degradability [12,38,39], which is consistent with the reduced PGP values observed for all diets containing EOC compared with the control diet. In addition, EOC is rich in polyphenols [12,38], which can have detrimental effects on ruminal fermentation, but the lack of effects of EOC on *c*, *lag* and AGPR values of the diets seems to preclude this hypothesis.

In accordance with that observed for gas production parameters, the inclusion of EOC up to 18% of the diet did not affect either total VFA production or the amount of AFOM at 8 h of fermentation (*p* ≥ 0.05). Exhausted olive cake can contain a considerable fraction of easily degradable sugars [12,38], which might help to explain the lack of differences among diets in these parameters at short incubation times, despite the EOC replacing highly degradable feeds (corn and wheat bran). Dietary inclusion of EOC modified the VFA pattern towards more acetate (*p* = 0.015) and less butyrate and branched-chain VFA (*p* ≤ 0.002) after 8 h of fermentation. In addition, NH_3_-N concentrations were linearly decreased (*p* = 0.001) by including increasing amounts of EOC in the diet, despite all diets being isonitrogenous. A high proportion of N in olive cake is usually bound to the ADF (ADIN) and therefore has a low level of availability [12,38,39]. The reduced NH_3_-N concentrations and branched-chain VFA concentrations observed for the EOC18 diet at 24 h of incubation also indicate lower CP degradability compared with the control, although NH_3_-N concentrations were adequate for in vitro ruminal microbial growth in all diets [40].

The lack of differences among diets in both total VFA production and AFOM (*p* ≥ 0.05) was also observed after 24 h, indicating that the use of EOC as a feed ingredient did not reduce diet fermentation. Compared with the control diet, the fermentation of the EOC18 diet resulted in greater propionate proportions, which could be due to the presence of sugars in the EOC. 

Recently, Aguilera and Molina-Alcaide [41] observed that the inclusion of high levels of crude olive cake (40% of the diet) reduced the enteric CH_4_ emissions in Segureña sheep. As reported by others [36] a high content of polyphenols in EOC might reduce the in vitro methane production, but no effects were detected in our study at any incubation time (*p* ≥ 0.05). The moderate content in total soluble polyphenols of the EOC used in our study (Table 1) and the lower dietary inclusion levels compared with those used by Aguilera and Molina-Alcaide [41] could explain the controversial results obtained in the different studies.

### 3.3. Fermentation of Diets including the Exhausted Olive Cake and Tomato Pomace Mixture

In agreement with previous reports [5,42] the TP sample used in our study was rich in NDF and had a relatively high content of CP and EE (Table 1). The EE content of TP might limit its inclusion level in ruminant diets [14], but the EOCTP mixture had lower EE content to prevent any negative effect on ruminal fermentation. 

As shown in Table 5, PGP linearly decreased by including increasing quantities of EOCTP in the diet (*p* = 0.009), which can be due to the low DM degradability of EOC, as the observed reduction in PGP numerically was lower for EOCTP (Table 5) compared with EOC (Table 4). 

The use of EOCTP in the diet increased linearly the gas production rate (*p* = 0.050) possibly due to the presence of easily fermented fractions in TP (i.e., sugars) [5], as this effect was not observed by using only EOC (Table 4). However, both *lag* and AGPR were unaffected (*p* ≥ 0.05) by the use of EOCTP as a feed ingredient.

Similarly to that observed for EOC, there were no effects of EOCTP (*p* ≥ 0.05) in total VFA production and AFOM at any incubation time, indicating that ruminal fermentation was not impaired. A previous in vitro study [5] showed that using up to 18% of TP in a diet for fattening ruminants increased both total VFA production and AFOM and decreased the acetate/propionate ratio. However, only subtle changes in molar proportions of the main VFA were observed in our study, as the one effect detected was a linear increase (*p* = 0.049) in acetate proportion at 8 h of incubation as the amount of EOCTP in the diet increased.

On the other hand, increasing the level of EOCTP in the diet linearly reduced the proportion of branched-chain VFA at both incubation times (*p* ≤ 0.024), and the NH_3_-N concentrations at 24 h of incubation (*p* = 0.003). These effects indicate a reduction in CP degradation, and this was already observed when using EOC alone (Table 4), but including TP in the EOCTP mixture probably further contributed. The amino acid profile of TP is similar to that of soybean meal [43] but its CP degradability is lower [42,44], probably due the high proportion of N bound to the fiber that can increase due the high temperatures used during tomato processing [5]. In fact, Marcos et al. [5] reported a lineal decrease in both branched-chain VFA proportions and NH_3_-N concentrations in 24 h in vitro incubations of diets for fattening ruminants including up to 18% of TP.

Finally, the inclusion of EOCTP in the diet had no effect on CH_4_ production at any time (*p* ≥ 0.05) and consequently no differences among diets in the CH_4_/VFA ratio were observed (*p* ≥ 0.05). In agreement with our results, Marcos et al. [5] reported no changes in the CH_4_ production or the CH_4_/VFA ratio when diets containing increasing amounts of TP were fermented in vitro. These results contrast with other studies reporting that the inclusion of tomato fruit wastes in the diet reduced the CH_4_ emissions in goats [6,45,46,47]. As discussed by Marcos et al. [5] the compounds responsible for the antimethanogenic effects of tomato fruits are still unknown, but it is possible that they are lost or inactivated during tomato processing.

### 3.4. Comparison of All Experimental Diets

**Figure 1:**
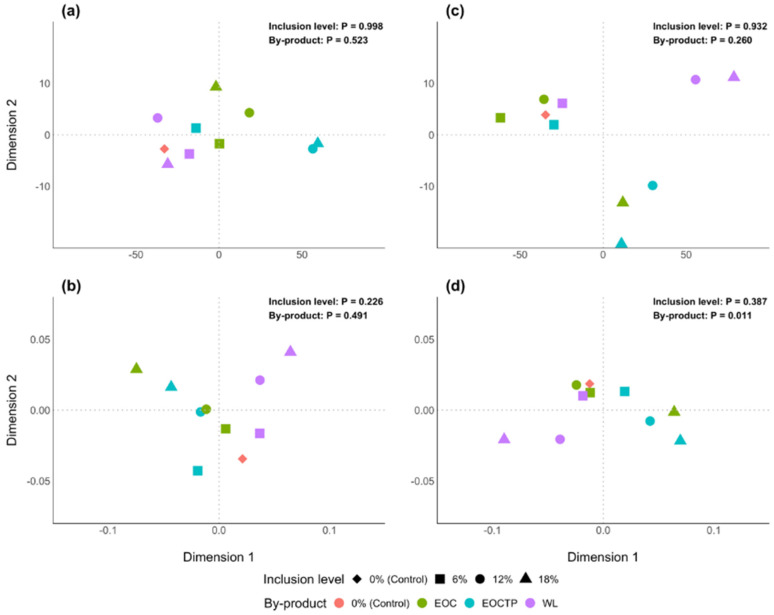
Multidimensional scaling comparison of experimental diets (each point represents an experimental diet). The axes values are arbitrary as in the orientation of the plot. Similarities (more similar diets are closer) among diets of ruminal fermentation parameters (total VFA and CH_4_ production, NH_3_ concentration and AFOM) and VFA profile (proportions of acetate, propionate, butyrate and branched-chain VFA) at 8 h ((**a**,**b**), respectively) and 24 h of in vitro incubation ((**c**,**d**), respectively). The *p* values of the MANOVA are also shown.

A MANOVA analysis was conducted to compare all diets and the results are shown in Figure 1. No differences (*p* ≥ 0.05) were observed among either by-products or inclusion levels after 8 h of incubation in ruminal fermentation parameters (VFA and CH_4_ production, NH_3_-N concentrations, and AFOM) or VFA profile. On the contrary, after 24 h of incubation, differences (*p* = 0.011) among by-products in the VFA profile were detected. It can be clearly noticed that diets including 6% of any by-product and the EOC12 diet had a similar fermentation to the control diet after 24 h, but the rest of diets were more distant.

The fermentation of the diets including either 18% of EOC or 12 and 18% of EOCTP resulted in greater propionate proportions but the extent of fermentation was lower, probably due to the lower fiber and CP degradation [34,39]. However, including more than 6% of WL in the diet had a negative impact on ruminal fermentation and this effect was more pronounced when reaching the 18% inclusion level.

## 4. Conclusions

The by-products evaluated in this study can be included up to 6% in diets for fattening ruminants with no negative effects on ruminal fermentation. Exhausted olive cake and its mixture with TP are fibrous ingredients that can be included up to 18% of the diet without decreasing the extent of fermentation but can cause a decrease in protein degradability. In contrast, the use of 12 or 18% of WL reduced the extent of fermentation. Information on the effects of WL on ruminal fermentation is scarce and more studies are needed to effectively determine their potential effects when used as feed ingredients for ruminants.

## Figures and Tables

**Table 1:** Chemical composition (g/kg dry matter (DM) unless stated otherwise) of by-products and feedstuffs used to formulate the experimental diets.

	Dry Matter (g/kg)	Organic Matter	Crude Protein	Neutral Detergent Fiber	Acid Detergent Fiber	Ether Extract	Total Soluble Polyphenols
By-product							
Wine lees (WL)	932	936	125	311	249	43.0	34.1
Exhausted olive cake (EOC)	823	830	90.6	565	432	31.3	23.5
Tomato pomace (TP)	960	967	163	572	446	95.2	3.47
EOC:TP mixture (1:1) ^1^	892	899	127	569	439	63.3	13.5
Conventional feedstuffs							
Barley	918	973	124	227	52.3	31.6	-
Wheat	900	982	153	147	29.2	22.8	-
Corn	871	988	68.1	98.2	20.6	41.3	-
Soybean meal	897	924	506	145	77.4	29.0	-
Wheat bran	891	946	187	417	127	55.5	-
Barley straw	974	937	37.3	787	432	16.9	-

^1^ Chemical composition calculated from analysed composition of OC and TP. Content in total soluble polyphenols of conventional feeds was not determined.

**Table 2:** Ingredients and chemical composition of experimental diets containing increasing quantities (0 (Control), 6, 12 and 18%; fresh matter basis) of wine lees (WL), exhausted olive cake (EOC), or a 1:1 mixture of EOC and tomato pomace (EOCTP).

Item	Control	WL6	WL12	WL18	EOC6	EOC12	EOC18	EOCTP6	EOCTP12	EOCTP18
Ingredient (g/kg fresh matter)										
Barley	335	335	335	335	335	335	335	335	335	335
Corn	252	222	192	162	240	228	210	252	252	252
Wheat	130	130	130	130	130	130	130	130	130	130
Barley straw	120	100	80.0	60.0	90.0	60.0	30.0	90.0	60.0	30.0
Soybean meal	90.0	80.0	70.0	60.0	90.0	90.0	90.0	78.0	68.0	57.0
Wheat bran	48.0	48.0	48.0	48.0	30.0	12.0	-	33.0	16.0	-
WL	-	60.0	120	180	-	-	-	-	-	-
EOC	-	-	-	-	60.0	120	180	-	-	-
EOCTP	-	-	-	-	-	-	-	60.0	120	180
Calcium soap	15.0	15.0	15.0	15.0	15.0	15.0	15.0	12.0	9.0	6.0
Calcium carbonate	5.0	5.0	5.0	5.0	5.0	5.0	5.0	5.0	5.0	5.0
Mineral/vitamin premix	5.0	5.0	5.0	5.0	5.0	5.0	5.0	5.0	5.0	5.0
Chemical composition ^1^										
Dry matter	903	905	906	907	897	892	883	900	898	895
Organic matter	946	948	946	944	942	935	928	950	950	951
Crude protein	137	137	137	137	138	139	140	135	134	133
Neutral detergent fiber	246	245	244	243	247	248	251	249	252	255
Acid detergent fiber	100	104	107	111	106	113	121	108	117	125
Ether extract	44.9	45.6	46.4	47.1	44.9	45.0	45.1	44.3	43.7	43.1
Non structural carbohydrates	518	520	519	517	512	503	492	520	516	519

^1^ Calculated from analyzed composition of individual feed ingredients. All chemical fractions are expressed as g/kg dry matter, except dry matter (g/kg fresh matter). Non-structural carbohydrates were calculated as [1000 − (ash + crude protein + neutral detergent fiber + ether extract)].

**Table 3:** Gas production parameters and fermentation parameters after 8 and 24 h of in vitro fermentation of diets with increasing amounts (0 (Control), 6 (WL6), 12 (WL12) and 18 (WL18); fresh matter basis) of wine lees (WL) incubated in batch cultures of mixed rumen microorganisms ^1^.

Item	Control	WL6	WL12	WL18	SEM ^1^	*p*-Value	
						Lineal	Quadratic
**Gas production parameters ^2^**							
PGP (mL/g dry matter (DM))	336 ^b^	325 ^b^	312 ^ab^	304 ^a^	3.7	<0.001	0.729
*c* (%/h)	4.17	4.27	4.36	4.37	0.132	0.211	0.690
*lag* (h)	1.63	1.82	1.53	1.12	0.231	0.107	0.223
AGPR (mL/h)	9.16	8.95	8.88	8.88	0.352	0.588	0.772
**Fermentation parameters at 8 h incubation ^3^**							
Total VFA (mmol/g DM)	3.47	3.51	3.46	3.48	0.065	0.909	0.853
Molar proportions (mol/100 mol)							
Acetate	57.7 ^a^	58.3 ^b^	59.2 ^c^	59.9 ^d^	0.13	<0.001	0.414
Propionate	23.9 ^c^	23.6 ^c^	22.8 ^b^	22.0 ^a^	0.27	0.003	0.395
Butyrate	15.0	14.7	14.7	14.8	0.17	0.461	0.452
Branched-chain VFA	3.37	3.41	3.32	3.34	0.050	0.521	0.911
Acetate/Propionate (mol/mol)	2.46 ^a^	2.52 ^a^	2.64 ^b^	2.78 ^c^	0.030	<0.001	0.231
NH_3_-N (mg/L)	185	186	179	188	3.44	0.999	0.283
CH_4_ (mL/g DM)	18.5	16.8	17.4	17.6	0.90	0.604	0.315
CH_4_/VFA (mL/mmol)	5.34	4.79	5.00	5.07	0.250	0.599	0.239
AFOM (mg/g)	315	318	313	315	6.0	0.855	0.909
**Fermentation parameters at 24 h incubation**							
pH	6.71 ^a^	6.70 ^a^	6.74 ^ab^	6.76 ^b^	0.020	0.021	0.210
Total VFA (mmol/g DM)	6.82 ^b^	6.79 ^b^	6.59 ^a^	6.53 ^a^	0.081	0.017	0.849
Molar proportions (mol/100 mol)							
Acetate	58.6 ^a^	59.1 ^b^	59.7 ^c^	60.1 ^d^	0.14	<0.001	0.830
Propionate	19.4 ^c^	19.3 ^c^	18.6 ^b^	18.0 ^a^	0.19	0.003	0.358
Butyrate	17.5	17.2	17.3	17.3	0.13	0.403	0.340
Branched-chain VFA	4.40 ^a^	4.40 ^a^	4.38 ^a^	4.55 ^b^	0.040	0.061	0.079
Acetate/Propionate (mol/mol)	3.08 ^a^	3.14 ^a^	3.27 ^b^	3.39 ^c^	0.030	<0.001	0.344
NH_3_-N (mg/L)	284	286	289	289	5.43	0.534	0.863
CH_4_ (mL/g DM)	43.1	42.0	42.3	43.0	1.10	0.996	0.469
CH_4_/VFA (mL/mmol)	6.32	6.19	6.42	6.58	0.160	0.201	0.387
AFOM (mg/g)	625 ^c^	620 ^bc^	603 ^ab^	598 ^a^	7.0	0.011	0.869

^abcd^ Within each parameter, mean values with different superscripts differ (*p* < 0.05). ^1^ SEM: standard error of the mean. ^2^ PGP: Potential gas production; *c*: Fractional rate of gas production; *lag*: Time before starting gas production; AGPR: Average gas production rate. ^3^ VFA: Volatile fatty acids; branched-chain VFA: isobutyrate, isovalerate, and valerate; AFOM: Apparently fermented organic matter estimated from VFA production (see text for calculations).

**Table 4:** Gas production parameters and fermentation parameters after 8 and 24 h of in vitro fermentation of diets with increasing amounts (0 (Control), 6 (EOC6), 12 (EOC12), and 18 (EOC18); fresh matter basis) of exhausted olive cake (EOC) incubated in batch cultures of mixed rumen microorganisms.

Item	Control	EOC6	EOC12	EOC18	SEM ^1^	*p*-Value	
						Lineal	Quadratic
**Gas production parameters ^2^**							
PGP (mL/g dry matter (DM))	336 ^b^	321 ^a^	320 ^a^	309 ^a^	4.4	0.003	0.677
*c* (%/h)	4.17	4.43	4.26	4.34	0.101	0.471	0.459
*lag* (h)	1.63	1.89	1.71	1.58	0.261	0.788	0.491
AGPR (mL/h)	9.16	8.84	8.84	8.75	0.343	0.380	0.985
**Fermentation parameters at 8 h incubation ^3^**							
Total VFA (mmol/g DM)	3.47	3.61	3.60	3.55	0.045	0.193	0.168
Molar proportions (mol/100 mol)							
Acetate	57.7 ^a^	58.1 ^ab^	58.3 ^b^	58.4 ^b^	0.16	0.015	0.404
Propionate	23.9	23.8	23.8	24.2	0.20	0.347	0.150
Butyrate	15.0 ^b^	14.8 ^ab^	14.7 ^ab^	14.4 ^a^	0.09	<0.001	0.285
Branched-chain VFA	3.37 ^b^	3.30 ^b^	3.23 ^ab^	3.03 ^a^	0.052	0.002	0.253
Acetate/Propionate (mol/mol)	2.46	2.48	2.50	2.44	0.033	0.888	0.162
NH_3_-N (mg/L)	185 ^b^	184 ^b^	178 ^a^	173 ^a^	2.1	0.001	0.528
CH_4_ (mL/g DM)	18.5	19.1	18.7	18.5	0.45	0.899	0.424
CH_4_/VFA (mL/mmol)	5.34	5.28	5.19	5.21	0.113	0.333	0.832
AFOM (mg/g)	315	323	325	320	4.0	0.246	0.164
**Fermentation parameters at 24 h incubation**							
pH	6.71	6.70	6.71	6.71	0.013	0.850	0.673
Total VFA (mmol/g DM)	6.82	6.88	6.82	6.70	0.048	0.102	0.101
Molar proportions (mol/100 mol)							
Acetate	58.6 ^a^	58.7 ^ab^	58.9 ^b^	58.4 ^a^	0.13	0.265	0.043
Propionate	19.4 ^a^	19.3 ^a^	19.3 ^a^	20.1 ^b^	0.12	0.108	0.008
Butyrate	17.5	17.6	17.3	17.4	0.07	0.209	0.812
Branched-chain VFA	4.45 ^b^	4.38 ^b^	4.44 ^b^	4.10 ^a^	0.061	0.012	0.031
Acetate/Propionate (mol/mol)	3.08 ^b^	3.10 ^b^	3.10 ^b^	2.96 ^a^	0.031	0.014	0.013
NH_3_-N (mg/L)	284 ^b^	284 ^b^	287 ^b^	266 ^a^	2.3	<0.001	0.002
CH_4_ (mL/g DM)	43.1	44.5	43.8	43.0	0.75	0.808	0.213
CH_4_/VFA (mL/mmol)	6.32	6.46	6.39	6.41	0.101	0.655	0.596
AFOM (mg/g)	625	633	623	615	4.5	0.115	0.135

^ab^ Within each parameter, mean values with different superscripts differ (*p* < 0.05). ^1^ SEM: standard error of the mean. ^2^ PGP: Potential gas production; *c*: Fractional rate of gas production; *lag*: Time before starting gas production; AGPR: Average gas production rate. ^3^ VFA: Volatile fatty acids; branched-chain VFA: isobutyrate, isovalerate, and valerate; AFOM: Apparently fermented organic matter estimated from VFA production (see text for calculations).

**Table 5:** Gas production parameters and fermentation parameters after 8 and 24 h of in vitro fermentation of diets with increasing amounts (0 (Control), 6 (EOCTP6), 12 (EOCTP12) and 18 (EOCTP18); fresh matter basis) of a 1:1 mixture of exhausted olive cake and tomato pomace (EOCTP) incubated in batch cultures of mixed rumen microorganisms.

Item	Control	EOCTP6	EOCTP12	EOCTP18	SEM ^1^	*p*-Value	
						Lineal	Quadratic
**Gas production parameters ^2^**							
PGP (mL/g dry matter (DM))	336 ^b^	331 ^b^	328 ^ab^	316 ^a^	4.3	0.009	0.439
*c* (%/h)	4.17 ^a^	4.44 ^ab^	4.57 ^b^	4.55 ^ab^	0.124	0.050	0.265
*lag* (h)	1.63	1.84	1.81	1.60	0.264	0.722	0.356
AGPR (mL/h)	9.16	9.47	9.62	9.39	0.438	0.670	0.547
**Fermentation parameters at 8 h incubation ^3^**							
Total VFA (mmol/g DM)	3.47	3.52	3.70	3.70	0.122	0.147	0.872
Molar proportions (mol/100 mol)							
Acetate	57.7 ^a^	58.1 ^ab^	58.2 ^ab^	58.3 ^b^	0.18	0.049	0.618
Propionate	23.9	24.2	23.9	24.0	0.38	0.990	0.887
Butyrate	15.0	15.6	14.7	14.5	0.21	0.265	0.593
Branched-chain VFA	3.37 ^b^	3.21 ^ab^	3.22 ^ab^	3.13 ^a^	0.057	0.024	0.589
Acetate/Propionate (mol/mol)	2.46	2.44	2.47	2.46	0.044	0.852	0.985
NH_3_-N (mg/L)	185	181	185	184	4.4	0.987	0.742
CH_4_ (mL/g DM)	18.5	18.7	19.1	20.3	0.88	0.180	0.602
CH_4_/VFA (mL/mmol)	5.34	5.32	5.19	5.48	0.173	0.721	0.394
AFOM (mg/g)	315	318	335	335	11.5	0.164	0.907
**Fermentation parameters at 24 h incubation**							
pH	6.71 ^b^	6.71 ^b^	6.69 ^ab^	6.67 ^a^	0.011	0.019	0.524
Total VFA (mmol/g DM)	6.82	6.80	6.66	6.70	0.083	0.217	0.723
Molar proportions (mol/100 mol)							
Acetate	58.6	58.4	58.6	58.7	0.12	0.460	0.153
Propionate	19.4	19.7	19.7	19.9	0.24	0.279	0.726
Butyrate	17.5	17.6	17.6	17.4	0.14	0.580	0.510
Branched-chain VFA	4.40 ^b^	4.28 ^ab^	4.15 ^ab^	4.03 ^a^	0.087	0.010	0.986
Acetate/Propionate (mol/mol)	3.08	3.01	3.02	3.00	0.043	0.294	0.501
NH_3_-N (mg/L)	284 ^c^	282 ^bc^	269 ^ab^	258 ^a^	5.0	0.003	0.416
CH_4_ (mL/g DM)	43.5	45.5	43.0	43.5	0.83	0.764	0.270
CH_4_/VFA (mL/mmol)	6.32	6.70	6.45	6.48	0.104	0.634	0.133
AFOM (mg/g)	625	625	613	615	6.8	0.218	0.754

^abc^ Within each parameter, mean values with different superscripts differ (*p* < 0.05). ^1^ SEM: standard error of the mean. ^2^ PGP: Potential gas production; *c*: Fractional rate of gas production; *lag*: Time before starting gas production; AGPR: Average gas production rate. ^3^ VFA: Volatile fatty acids; branched-chain VFA: isobutyrate, isovalerate, and valerate; AFOM: Apparently fermented organic matter estimated from VFA production (see text for calculations).

## Data Availability

Not applicable.
